# Non-canonical dorsoventral patterning in the moth midge *Clogmia albipunctata*

**DOI:** 10.1186/s13227-017-0083-9

**Published:** 2017-11-13

**Authors:** Karl R. Wotton, Anna Alcaine-Colet, Johannes Jaeger, Eva Jiménez-Guri

**Affiliations:** 1 EMBL/CRG Research Unit in Systems Biology, Centre de Regulació Genòmica (CRG), The Barcelona Institute of Science and Technology (BIST), Dr. Aiguader 88, 08003 Barcelona, Spain; 2 Universitat Pompeu Fabra (UPF), Barcelona, Spain; 3 Present Address: Centre for Ecology and Conservation, College of Life and Environmental Sciences, University of Exeter, Penryn, Cornwall TR10 9EZ UK; 4 Present Address: Complexity Science Hub Vienna, Josefstädter Straße 39, 1080 Vienna, Austria

## Abstract

**Background:**

Bone morphogenetic proteins (BMPs) are of central importance for dorsal–ventral (DV) axis specification. They are core components of a signalling cascade that includes the BMP ligand decapentaplegic (DPP) and its antagonist short gastrulation (SOG) in *Drosophila melanogaster*. These components are very ancient, with orthologs involved in DV patterning in both protostomes and deuterostomes. Despite such strong conservation, recent comparative work in insects has revealed interesting differences in the way the patterning function of the DV system is achieved in different species.

**Results:**

In this paper, we characterise the expression patterns of the principal components of the BMP DV patterning system, as well as its signalling outputs and downstream targets, in the non-cyclorrhaphan moth midge *Clogmia albipunctata* (Diptera: Psychodidae). We previously reported ventral expression patterns of *dpp* in the pole regions of *C. albipunctata* blastoderm embryos. Strikingly, we also find ventral *sog* and posteriorly restricted *tkv* expression, as well as expanded polar activity of pMad. We use our results from gene knock-down by embryonic RNA interference to propose a mechanism of polar morphogen shuttling in *C. albipunctata*. We compare these results to available data from other species and discuss scenarios for the evolution of DV signalling in the holometabolan insects.

**Conclusions:**

A comparison of gene expression patterns across hemipteran and holometabolan insects reveals that expression of upstream signalling factors in the DV system is very variable, while signalling output is highly conserved. This has two major implications: first, as long as ligand shuttling and other upstream regulatory mechanisms lead to an appropriately localised activation of BMP signalling at the dorsal midline, it is of less importance exactly where the upstream components of the DV system are expressed. This, in turn, explains why the early-acting components of the DV patterning system in insects exhibit extensive amounts of developmental systems drift constrained by highly conserved downstream signalling output.

## Background

**Fig. 1:**
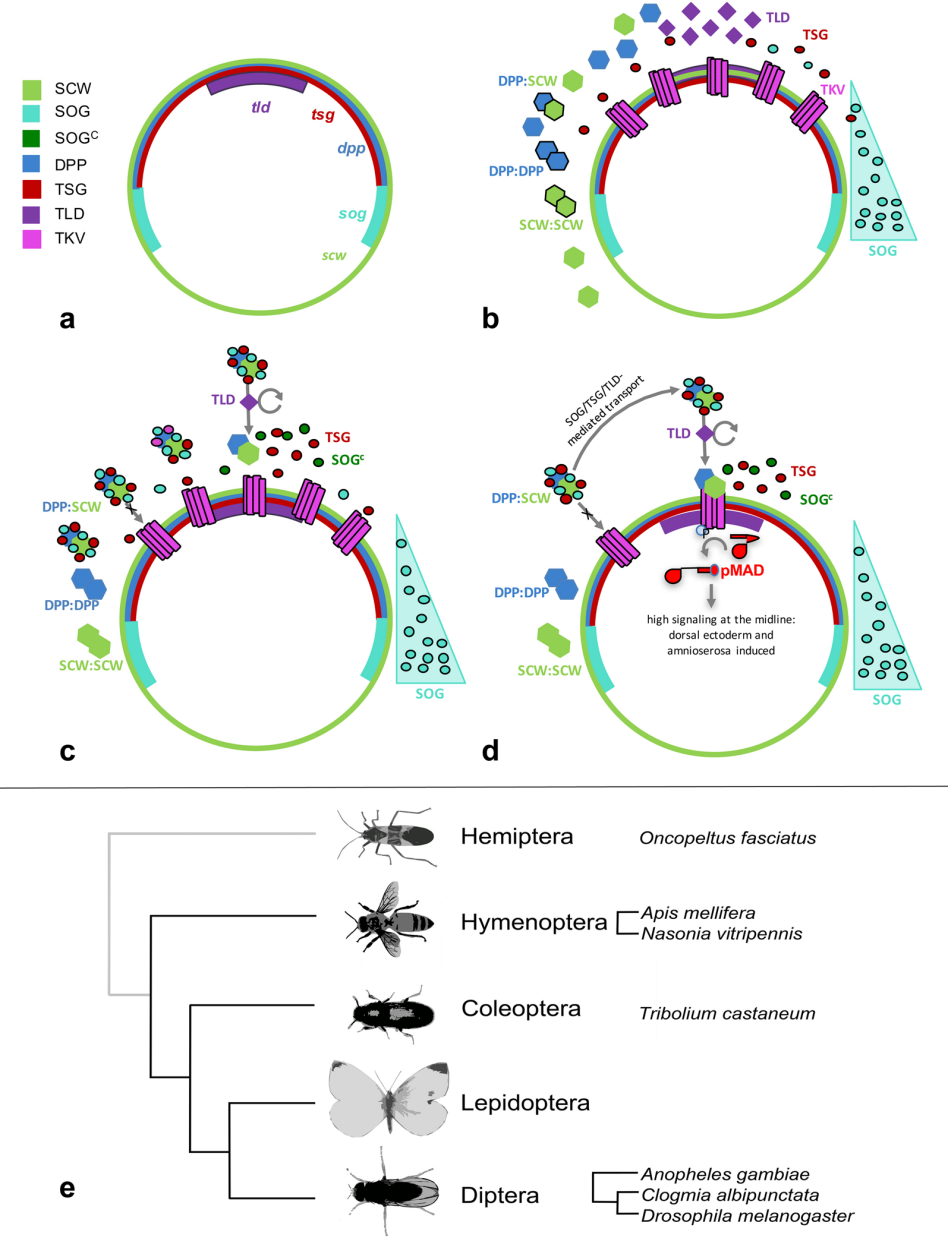
Schematic representation of the shuttling mechanism underlying DV patterning in *Drosophila melanogaster* (**a**–**d**) and phylogeny of species mentioned in the text (**e**). Circles in **a**–**d** represent transverse sections through blastoderm embryos. **a** mRNA expression patterns for the main components of the shuttling mechanism: green: *scw*; light blue: *sog*; blue: *dpp*; maroon: *tsg*; purple: *tld*. **b** Initial localisation of protein products. DPP and SCW form homo- and heterodimers. SOG diffuses towards the dorsal midline forming a concentration gradient (light blue triangle). Magenta: TKV. **c** Initial stages of the shuttling mechanism. BMP ligand dimers enter into the heteromeric DPP-SOG-SCW-TSG shuttling complex, which diffuses dorsally driven by the SOG gradient. The TLD protease cleaves SOG at the dorsal midline, releasing TSG and BMP dimers from the complex. SOG^c^ refers to cleaved SOG. **d** Released BMP ligand dimers bind their receptors, activating an intracellular signalling cascade mediated by MAD phosphorylation (pMAD) at the dorsal midline. pMAD translocates to the nucleus and induces target genes in a concentration-dependent manner, thereby inducing dorsal fate. **e** Phylogenetic tree showing the main groups of insects, and the species discussed in this paper. In black lines, holometabolous insects. Phylogeny based on Misof et al. [81]. See text for further details

Signalling molecules of the TGF-β family are widely conserved across the animal kingdom. They are key factors in developmental processes such as dorsal–ventral (DV) axis specification, appendage formation, patterning of the central nervous system, and cell proliferation (reviewed in [1]). Bone morphogenetic proteins (BMPs) are of particular interest for DV axis specification, because they determine dorsal embryonic tissues in those invertebrate species where they have been studied so far [2–5]. In the vinegar fly *Drosophila melanogaster*, for example, three BMP ligands are involved in development: they are encoded by *decapentaplegic* (*dpp*), *screw* (*scw*) and *glass bottom boat* (*gbb*) [6–8]. DPP and SCW proteins are required for DV axis specification, forming homo- and heterodimers to generate a robust, strictly localised, dorsal signalling gradient during the blastoderm stage of early development [9]. Extracellular BMP inhibitors encoded by *short gastrulation* (*sog*) and *twisted gastrulation* (*tsg*) bind DPP/SCW heterodimers generating a multimeric complex [9, 10] (Fig. 1a–c). The tolloid (TLD) protease cleaves this complex [11], liberating DPP/SCW. The resulting free ligand dimers attach to their receptors [encoded by *punt* (*put*), *saxophone* (*sax*), and *thick veins* (*tkv*)], which form transmembrane complexes [9, 12] (Fig. 1c). This binding event triggers an intracellular signalling process: once the ligand–receptor complex has been established, SAX and TKV phosphorylate Mothers against dpp (MAD). Phosphorylated MAD (pMAD) (associated with the signal transducer Medea) translocates to the nucleus (Fig. 1d) to activate or repress a number of target genes (such as *brinker*, *Dorsocross*, *tailup*, *twist*, *snail*, *pannier*, among others), whose localised regulation induces different tissue fates along the DV axis. In the DV patterning system, initially triggered by the Toll signalling pathway [13, 14], *dpp* is a key factor for the determination of dorsal tissues and acts as a morphogen for the specification of the dorsal ectoderm and the amnioserosa [6, 15]; see [9, 10] for a review.

In *D. melanogaster*, the heteromeric shuttling complex (DPP-SOG-SCW-TSG) transports the *dpp* protein ligand dorsally from the lateral regions of the broad *dpp* expression domain (Fig. 1). SOG is the component responsible for the diffusive transport of the complex from the ventral–lateral region, where *sog* is expressed, towards the dorsal region, where the concentration of SOG is low [9, 11, 16]. Once close to the dorsal midline, the complex is cleaved by TLD (Fig. 1c). This process of complex formation, diffusion, and subsequent local cleavage creates a sink for the shuttling complex, and DPP in particular, around the dorsal midline. High concentrations of SOG laterally ensure capture of DPP for transport. Two additional factors help to ensure that DPP signalling only occurs in a restricted dorsal range. First, *pnt* and *sax* are ubiquitously expressed, but *tkv* is restricted to dorsal regions, and all three receptors are needed for signalling to occur. Second, low concentrations of SOG dorsally make rebinding to DPP unlikely, which therefore remains free to attach predominantly to its receptors in this region (Fig. 1). A similar shuttling mechanism is proposed to be in place in vertebrates, although the DV polarity of the system is reversed in these organisms [17].

The function of the genes involved in this signalling cascade appears to be conserved in insects, and some components of the system are very ancient, controlling DV patterning in both protostomes and deuterostomes [18, 19]. Despite such strong conservation, recent work on holometabolan insects, including the mosquito *Anopheles gambiae* [20], the honey bee *Apis mellifera* [4], the jewel wasp *Nasonia vitripennis* [5, 21, 22], and the red flour beetle *Tribolium castaneum* [3, 23, 24], together with hemipteran milkweed bug *Oncopeltus fasciatus* has revealed some interesting differences in the way the patterning function of the system is achieved (see Fig. 1e for a phylogeny of the main species compared in this study). For example, one major difference between *D. melanogaster* and these insects is the absence of *scw*, a gene which originated recently within the lineage of the cyclorrhaphan flies via tandem duplication of *gbb* [25]. Other differences include the expression of key genes like *sog* and *dpp*, and the implications these altered patterns have for the signalling pathway. Hence, the DV patterning system in insects provides an interesting case study for how evolutionary changes in the distribution and timing of gene expression affect an ancient patterning system based on morphogenetic gradients.

In this paper, we characterise the expression patterns of the principal components of the DV patterning system and its downstream targets in *Clogmia albipunctata* (Diptera: Psychodidae). *C. albipunctata* is a member of the Psychodomorpha, a basally branching lineage of dipterans [26, 27]. It is an emerging model for evolutionary and developmental studies (see [25, 26, 28–32]). We have previously reported that a single copy *dpp* is expressed in the ventral pole regions of the blastoderm embryo [25]. This species lacks *scw,* while a single copy of *gbb* is expressed at the blastoderm stage in a large central domain, excluding the poles of the embryo [25]. In addition, we assay gene expression in embryos depleted of *dpp*, *gbb,* and *sog* transcripts via RNA interference (RNAi). We compare the expression patterns observed in *C. albipunctata* to those in other insects (*A. mellifera*, *N. vitripennis*, *T. castaneum, A. gambiae,* and *O. fasciatus*). Our comparative analysis and RNAi investigation suggest specific mechanisms for the establishment of the DV signalling morphogen gradients and indicate evolution of DV patterning by extensive developmental system drift [33, 34].

## Methods

### Gene identification, cloning, and synthesis of RNA constructs

We searched the early embryonic transcriptome of *C. albipunctata* ([26]; http://diptex.crg.es) using BLAST with sequences from *D. melanogaster* retrieved from GenBank. Gene identity was confirmed via reciprocal BLAST against the *D. melanogaster* genome. PCR primers were designed from transcriptome sequences (Table 1). Amplified sequences for *C. albipunctata* have been deposited in GenBank (see Table 1 for accession numbers). Fragments were cloned into the PCRII-TOPO vector (Invitrogen) and used to make DIG or FITC-labelled riboprobes for whole-mount in situ hybridisation, as well as double-stranded RNA constructs for RNAi. Constructs for RNAi against *C. albipunctata dpp* and *gbb* were synthesised from clones of sequences KC810051 and KC810052, respectively, and for *sog* from MF457413. We utilised primers containing a T7 promoter at their 5′ end to amplify a DNA template. This template was then purified and used to simultaneously synthesise sense and antisense RNA strands using T7 RNA polymerase. The residual DNA was then digested using RNAse-free DNAse before being extracted three times using phenol/chloroform/isoamylalcohol followed by precipitation in ethanol. The resulting pellet was washed in 70% ethanol before being resuspended in injection buffer (100 mM NaPO4, pH 6.8; 5 mM KCl). RNA was then annealed to form double-stranded (ds) RNA in a thermocycler by cycling at 94 °C for 40 s, then reducing the temperature by − 0.1 °C per cycle for 750 cycles. dsRNA was checked on an agarose gel. We obtained dsRNAs of the following length: *dpp,* 1550 bp; *gbb,* 1640 bp; and *sog,* 1460 bp. Constructs were quantified and, if necessary, diluted and reannealed to a concentration of 4–6 µM before being stored at − 20 °C. Before each use dsRNA was defrosted and centrifuged for 15 min at 12,000*g* in a desktop centrifuge to remove and debris to prevent clogging of the microinjection needle.

**Table 1:** Primers used and GenBank accession numbers for the genes cloned in this study

Gene	Primer forward 5′–3′	Primer reverse 5′–3′	Acc. num.
*brk*	AATTGCCAGCAACAGAGCTT	GTCTGGTGGTACTGGGGATG	MF457412
*Doc*	TCAGGCAATTATGGTATTAGGC	CGTGTTTTCTCCTCGTTAGCA	MF457411
*ind*	CGATCGTCCTTTTTCATTGG	AAACCCCCAATATCCTGAAAA	MF457416
*msh*	CCAGGTGAAGATCTGGTTCC	TTCTTTTTCAGCACCACCCTA	MF457420
*pnr*	TCAAGAGCCGACGGTAATCT	GGTGGATATGGCTCCACAAT	MF457418
*sna*	AGGCATCCTCCTAATGGCTAA	CAGCTGCAAAAACTGTGACAA	MF457419
*sog*	TGTGACATGCTGATCGAGTACA	ACCAATGGTCTAGCTTGGTTGT	MF457413
*tkv*	GCGCTACATAGTCTTCGCACT	AAACTTTAACCGTTGCCCAAG	MF457410
*tld*	ACTGCCGCACGGATTATCT	TTGATTGCACACTCGTCCAT	MF457414
*tsg*	TCGAACAAACAACAACAAAACA	AAAATGGAGGGAATGGCTAAA	MF457409
*twi*	GCAAAATCCAGACCCTCAGA	CAGCCCGTCGGAATAGATAA	MF457417
*vnd*	TCCCCTTAATCCTCAACGTG	CAGAGCCAACAACAGCTGAA	MF457415

Gene names: *brk: brinker; Doc: Dorsocross; ind: intermediate neuroblast defective; msh: muscle segment homeobox; pnr: pannier; sna: snail; sog: short gastrulation; tkv: thick veins; tld: tolloid; tsg: twisted gastrulation; twi: twist; vnd: ventral nervous system defective*

### Embryo collection and fixation

Wild-type and RNAi-treated embryos of *C. albipunctata* were collected at blastoderm and post-gastrulation stages as described previously in [28]. For whole-mount in situ hybridisation, embryos were heat-fixed using a protocol adapted from [35]. In brief, embryos were dechorionated at the desired developmental stage by submerging in 25% bleach for 45 s. They were then heat-fixed for 20 s in boiling fixing solution (0.7% NaCl; 0.05% Tween-20); heat fixation was stopped by adding water. These embryos then underwent a second fixation with formaldehyde (5%) and PBS/methanol. Devitellinisation was achieved in 50% heptane–methanol by vigorous shaking for 20 s, and embryos were preserved in methanol. For RNAi treatment, embryos were dechorionated by hand and fixed as described above. For immunostaining, embryos were fixed using a solution of PEMS/methanol, formaldehyde, and heptane for 25 min at room temperature [36], followed by devitellinisation and storage as described above.

### Whole-mount in situ hybridisation and immunohistochemistry

Whole-mount in situ hybridisation was performed as described previously in [26]; and references therein. In brief, after rehydration for 5 min in 50% MetOH-PBT followed by PBT washes, embryos were permeabilised by proteinase K treatment (8 mg/ml PBT) for 7 min at room temperature (RT), followed by 25-min refixation (5% formaldehyde in PBT). After washes and prehybridisation for 90 min at 56 °C, hybridisation was carried out overnight at 56 °C with a labelled probe at a concentration of 0.5–1 ng/μl. After washes and blocking in 5% heat-inactivated goat serum, antibody antidigoxigenin or fluorescein conjugated with alkaline phosphatase (Sigma) was used at 1:2000 for 2 h at RT followed by washing overnight. Staining was achieved using NBT/BCIP or fast red, and embryos were counterstained with DAPI and mounted in 70% glycerol. Enzymatic immunodetection of pMAD was done using a cross-reactive monoclonal anti-Smad3 antibody (phosphor S423 + S425) [EP823Y] (Abcam, UK, cat number ab52903) at a 1:10 dilution following the protocol described previously in [30]. In brief, after blocking in PBTB, embryos were incubated with the antibody at a concentration 1:10 overnight at 4 °C. Embryos were incubated in secondary antibodies (antirabbit conjugated with alkaline phosphatase) for 2 h at RT. Staining was achieved using NBT/BCIP, counterstaining with DAPI, and embryos were mounted in 70% glycerol.

### RNA interference (RNAi)

RNAi treatment was carried out based on protocols established in other dipteran species [37–39]. In brief, activated embryos were allowed to develop for 2 h before submerging them in 25% bleach for 10 s to weaken the chorions. Embryos were then rinsed in water under a tap for 1 min before being aligned on a microscope slide against a glass capillary (Hilgenberg 1421602 65 mm × OD 0.25 mm) and covered with a 3:1 mixture of 10:27 halocarbon oil. Embryos were injected while maintaining a constant flow of liquid to avoid blocking of the needle. Aluminosilicate (rather than borosilicate) capillaries were pulled in Sutter P-97 Flaming/Brown Micropipette Puller to produce needles better able to penetrate with the hard extraembryonic membranes of *C. albipunctata* without breaking. After injection embryos were allowed to develop for 7 h before being sprayed into a mesh disc with water from a squeeze bottle. The mesh and embryos were then transferred into a 50-ml tube to be fixed and collected as previously described [35]. We injected buffer only as a negative control, along with in situ hybridisation staining for depleted transcripts. *sog* dsRNA was injected four independent times, with a survival to blastoderm stage of 22%, obtaining approximately 300 embryos, *dpp* dsRNA was injected three times, with survival to blastoderm of 15%, leading to approximately 150 embryos, and *gbb* dsRNA was injected two times, with survival to blastoderm stage of 18%, obtaining around 120 embryos. For cuticle preparations, embryos were allowed to develop until first larval instar stages and were then manually extracted from the eggshell under oil using tungsten needles. Additional oil was dissolved in heptane, and the soft tissue removed in an acetic acid/glycerol solution for one day. Cuticles were then transferred to a slide in Hoyers medium and lactic acid (2:1) and covered with a cover slip; residual soft tissue was removed by placing at 65 °C overnight. Only dsRNA-injected cuticles presented abnormal phenotypes.

### Cryosections of stained embryos

Embryos were embedded in optimal cutting temperature (OCT) media and preserved at − 80 °C. Using a CM3050 S cryostat (Leica, Germany), 20-μm transversal sections were obtained.

## Results

### Expression patterns of the DV patterning system components and its downstream targets in *C. albipunctata*

We examined the expression patterns of the major components of the BMP signalling cascade comprising the DV patterning system as well as its core signalling output and key downstream targets in blastoderm and post-gastrulation embryos of the moth midge *C. albipunctata*. Where necessary, we utilised double in situ hybridisation to establish the relative position of expression patterns with regard to each other.

#### Identification of the major components of the DV patterning system

We previously isolated single copies of *dpp* and *gbb* from an early embryonic transcriptome [26]. We used the same genomic resource to identify other members of the DV patterning system in *C. albipunctata* (Table 1) and assigned orthology by reciprocal BLAST. Only *Doc* had more than one copy in the transcriptome. Two paralogues were found that appear to have arisen from an independent duplication within the lineage leading to *C. albipunctata* because they branch together in a phylogenetic tree, (http://phylomedb.org/?q=search_tree&phyd=174&seqid=CAL_comp7148_c0_seq1). We used the longer sequence to design primers. No duplicates of any other DV genes analysed here were present in the transcriptome. Cloned sequences were deposited in GenBank with the accession numbers as listed in Table 1.

#### Expression of the major components of the DV patterning system

**Fig. 2:**
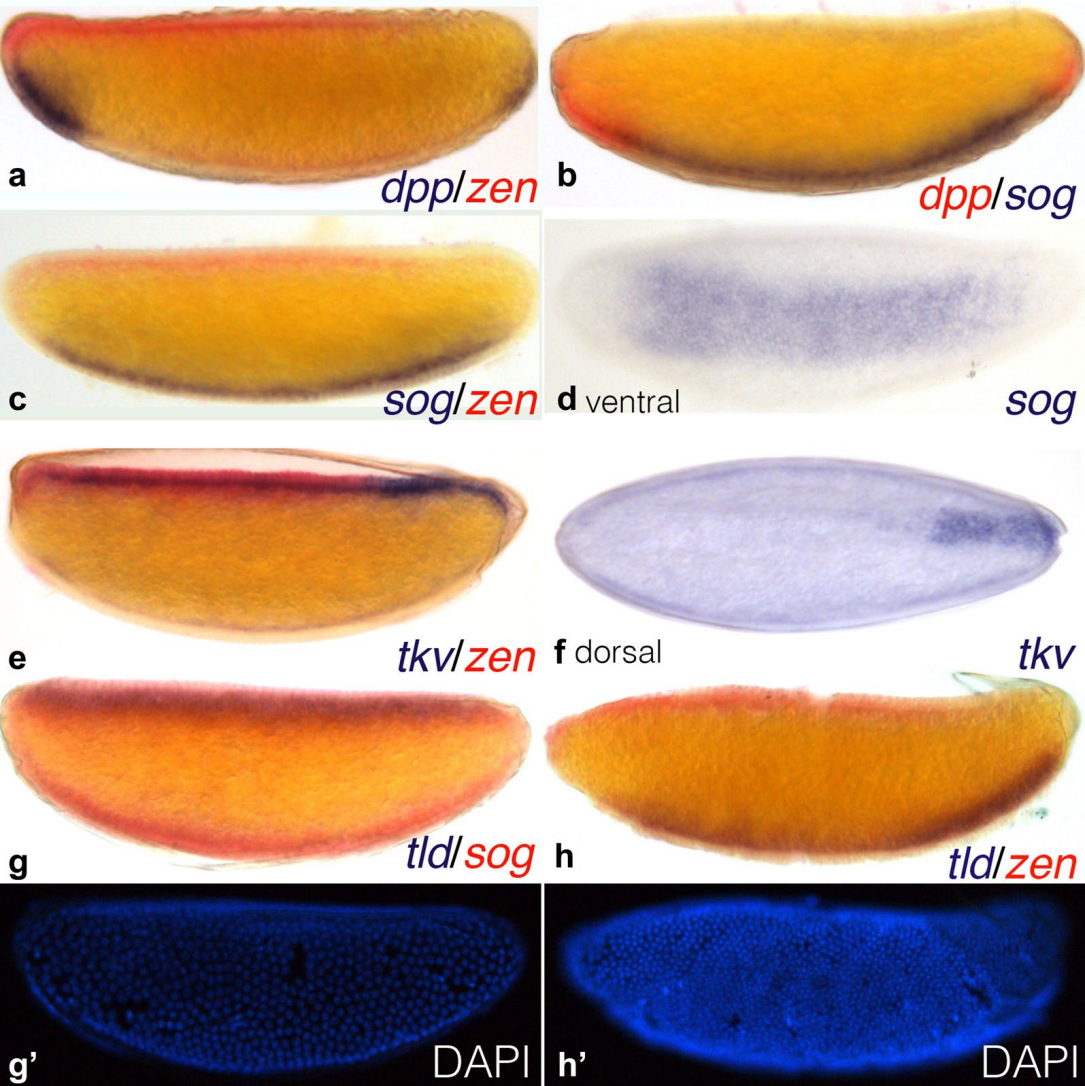
Expression of the main components of the BMP signalling cascade comprising the DV patterning system. Panels show whole-mount in situ hybridisation of *C. albipunctata* blastoderm-stage embryos. All embryos shown in lateral view with anterior to the left, dorsal to the top, except **d** (ventral view) and **f** (dorsal view). Stains as indicated in panels: **a** *dpp* (blue)/*zen* (red); **b** *dpp* (red)/*sog* (blue); **c** *sog* (blue)/*zen* (red); **d** *sog* (blue); **e** *tkv* (blue)/*zen* (red); **f** *tkv* (blue); **g** *tld* (blue)/*sog* (red); **h** *tld* (blue)/*zen* (red); **g′**, **h′** DAPI nuclear counterstaining of embryos in **g**, **h**. Counterstains were used for staging as described in [30]: **f**/**f′** is at cleavage stage 11 (C11), and **g**/**g′** is at cleavage stage 14 (C14)

In previous work, we detected *dpp* transcripts in two ventral domains within the anterior and posterior pole regions of the embryo [25] (Fig. 2a, blue; Fig. 2b, red). Using double in situ hybridisation, we detect expression of the *dpp* inhibitor *sog* on the ventral side of the embryo, excluded from the pole regions (Fig. 2b–d, blue; Fig. 2g, red). This pattern abuts and complements that of *dpp* with only a slight overlap, predominantly in the anterior region (Fig. 2b). We detect expression of the receptor *tkv* in a posterior domain at the dorsal midline (Fig. 2e, f, blue). We also cloned the other two *D. melanogaster* DPP receptors, *punt* and *saxophone. Unfortunately*, we could not detect any clear expression patterns of these genes by in situ hybridisation in *C. albipunctata* embryos. Finally, the protease *tld* is expressed dorsally in early blastoderm embryos (Fig. 2g, blue; 2g′), but there is a shift of expression towards the ventral side in late blastoderm embryos (Fig. 2h, blue; 2h′). This shift between dorsal and ventral expression occurs between C11 and C12 (*n* = 62). While all blastoderm embryos before C11 have dorsal *tld* expression, almost 40% of C11 embryos and about 80% of those at C12 show ventral expression, and all embryos after C12 have ventral expression (not shown).

#### Spatial distribution of BMP signalling outputs

**Fig. 3:**
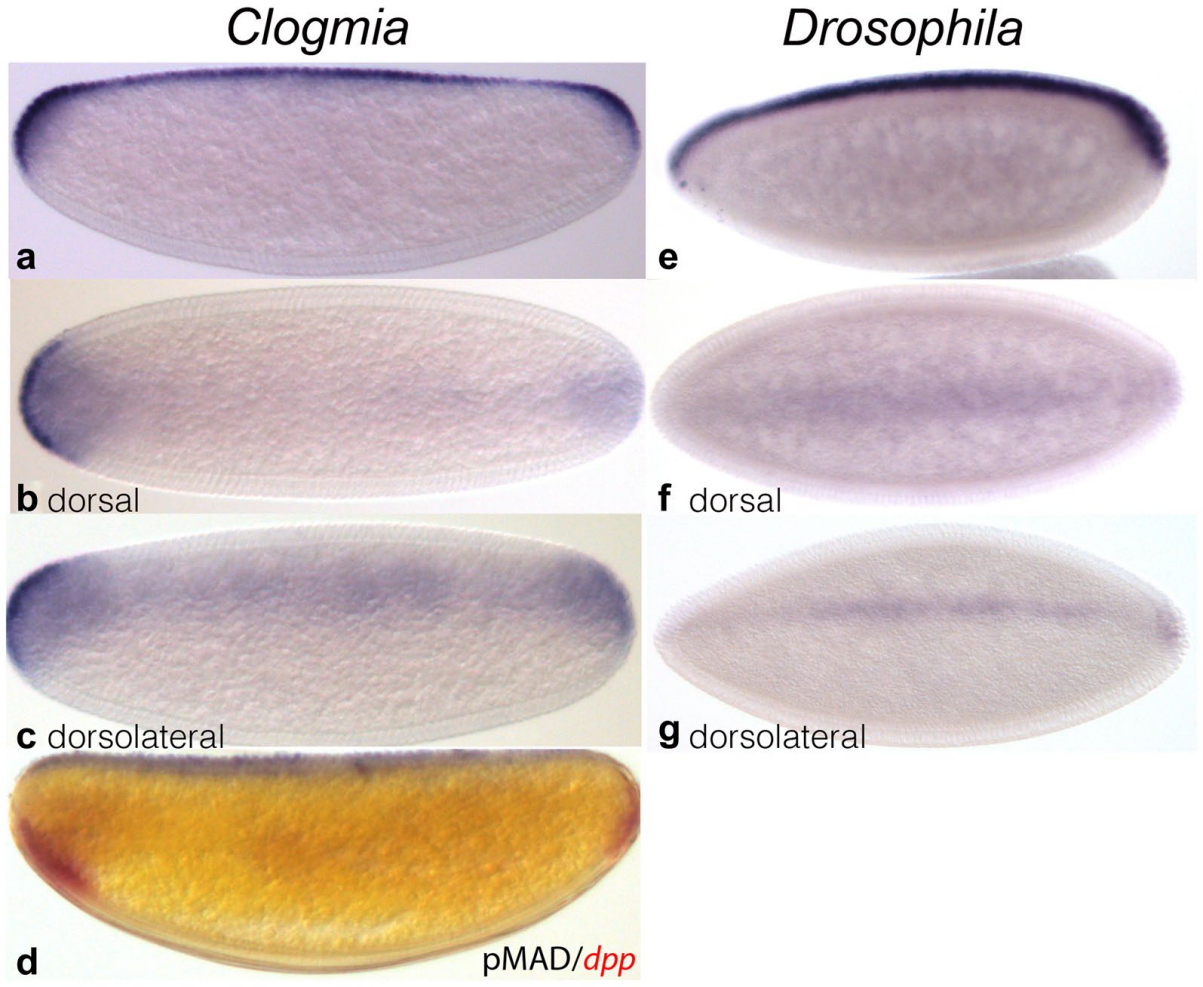
Localisation of DPP signalling output. Panels show immunostaining against pMAD protein in *C. albipunctata* (**a**–**d**) and *D. melanogaster* (**d**–**f**) blastoderm embryos, as well as *dpp* in situ hybridisation (**d**, red). Embryos in **a**, **d**, **e** shown in lateral view (anterior is to the left, dorsal to the top); embryos in **b**, **f** shown in dorsal view (anterior to the left); embryos in **c**, **g** are aligned dorsolaterally (anterior to the left). Blue stain: immunostaining against pMAD; red stain: in situ hybridisation for *dpp*

We detected BMP signalling activity by immunostaining against pMAD in blastoderm-stage embryos of *C. albipuncata* using a cross-reactive antibody (see Materials and Methods). We found pMAD to be localised in a contiguous narrow domain along the dorsal midline (Fig. 3a–d, blue). This domain expands laterally at both poles of the embryo (Fig. 3b, c), an effect not seen in *D. melanogaster* (Fig. 3f, g), if a subtle posterior expansion has been described before [40]. The expanded dorsal–polar domains of pMAD are similar in size and antero-posterior extent to the more ventral expression domains of *dpp* in these regions (Fig. 3d). Therefore, both expression patterns seem to mirror each other in the polar regions (Fig. 3b–d).

#### Downstream targets of BMP signalling

**Fig. 4:**
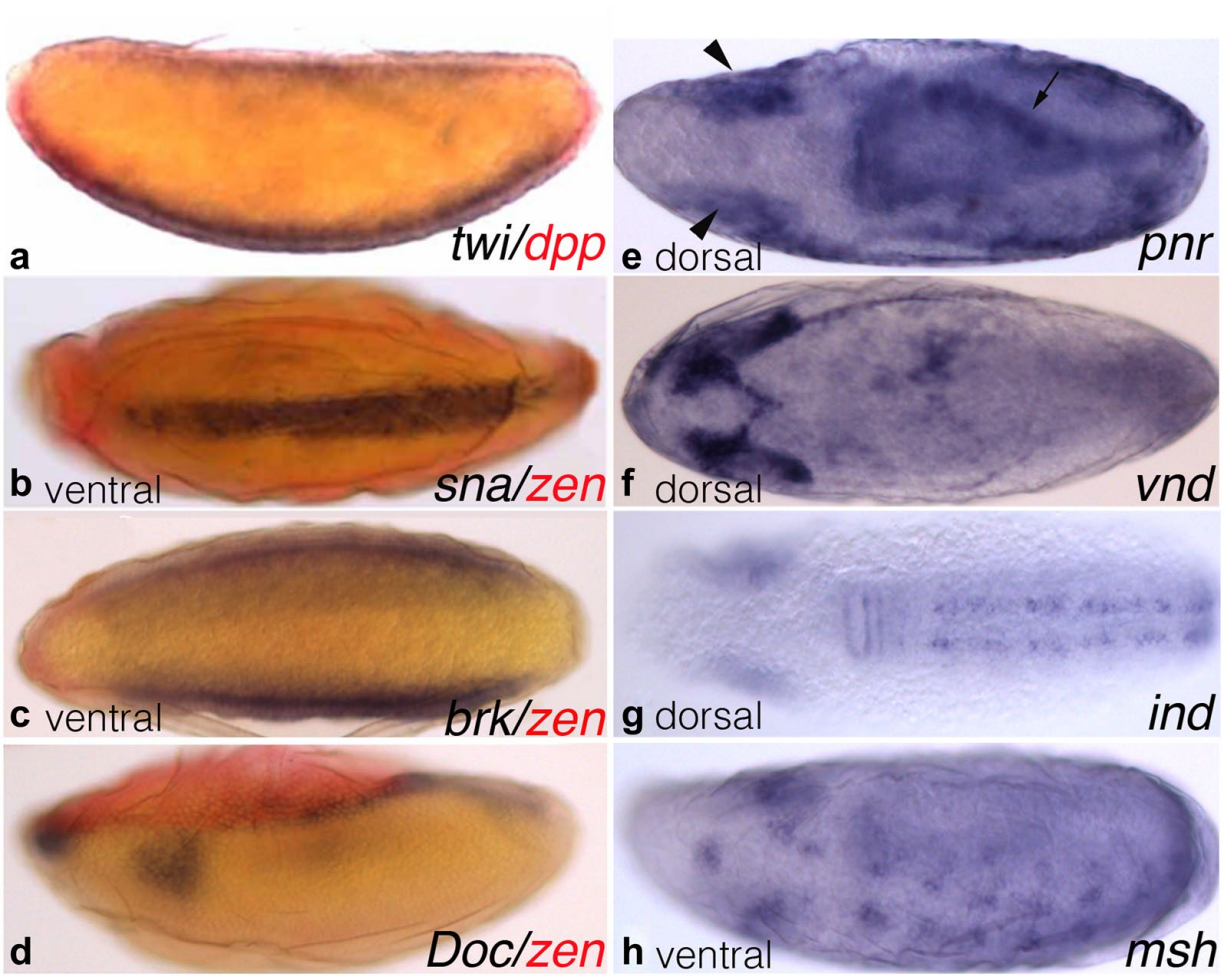
Expression of DV target genes. Panels show whole-mount in situ hybridisation of *C. albupunctata* blastoderm-stage (**a**–**d**) and germband-stage (**e**–**h**) embryos. Embryos in a, d shown in lateral view (dorsal to the top); **b**, **c**, **h** in ventral view, **e**–**g** in dorsal view (anterior is to the left in all panels). Stains as indicated in panels: **a** *twi* (blue)/*dpp* (red); **b** *sna* (blue)/*zen* (red); **c** *brk* (blue)/*zen* (red); **d** *Doc* (blue)/*zen* (red); **e** *pnr* (blue); **f** *vnd* (blue); **g** *ind* (blue); **h** *msh* (blue)

We characterise the expression patterns of downstream targets of *D. melanogaster* BMP signalling in *C. albipunctata*. In *D. melanogaster,* low levels of BMP signalling coincide with the ventral activation of *twist* (*twi*) and *snail* (*sna*) defining the mesodermal region of the embryo [41, 42]. Similarly, in *C. albipunctata*, *twi* and *sna* are expressed in domains along the ventral midline of the blastoderm embryo (Fig. 4a, b). In *D. melanogaster, brinker* (*brk*) is a transcriptional repressor of DPP target genes negatively regulated by DPP signalling activity [43]. In *C. albipunctata*, we find that *brk* is expressed in two ventral–lateral domains (Fig. 4c). In *D. melanogaster*, *pannier* (*pnr*) and *Dorsocross* (*Doc*) are activated by high levels of DPP signalling activity along the dorsal midline [2, 44, 45]. They are involved dorsal closure [46], and in the specification of the amnioserosa [45], respectively. In *C. albipunctata,* we detect *Doc* expression in a dorsal domain (excluding the serosa) and a head stripe during the blastoderm stage (Fig. 4d). *C. albipunctata pnr* is not unambiguously detectable until after gastrulation; it then shows expression at the dorsal ectoderm ridge (Fig. 4e, arrow) and in the head lobes (Fig. 4e, arrowheads). In addition, we looked at the laterally expressed markers of the neurogenic ectoderm, the columnar genes *vnd*, *ind*, and *msh* [47–49]. In *C. albipunctata*, these genes are only detectable after gastrulation, in expression patterns restricted to tissues of the nervous system. We see no obvious DV polarity in the expression of *vnd*, with its broad anterior head domain (Fig. 4f). *ind* has a ventral expression pattern in two spotted stripes along the antero-posterior axis (Fig. 4g). *msh* is expressed in a similar pattern, dorsal to *ind* (Fig. 4h). All in all, the expression patterns of these target genes are very similar to those found in *D. melanogaster* and other insects.

### Functional characterisation of the DV patterning system in *C. albipunctata*

To functionally characterise the DV patterning system in *C. albipunctata,* we knocked down expression of *dpp, gbb,* and *sog* using RNA interference (RNAi).

**Fig. 5:**
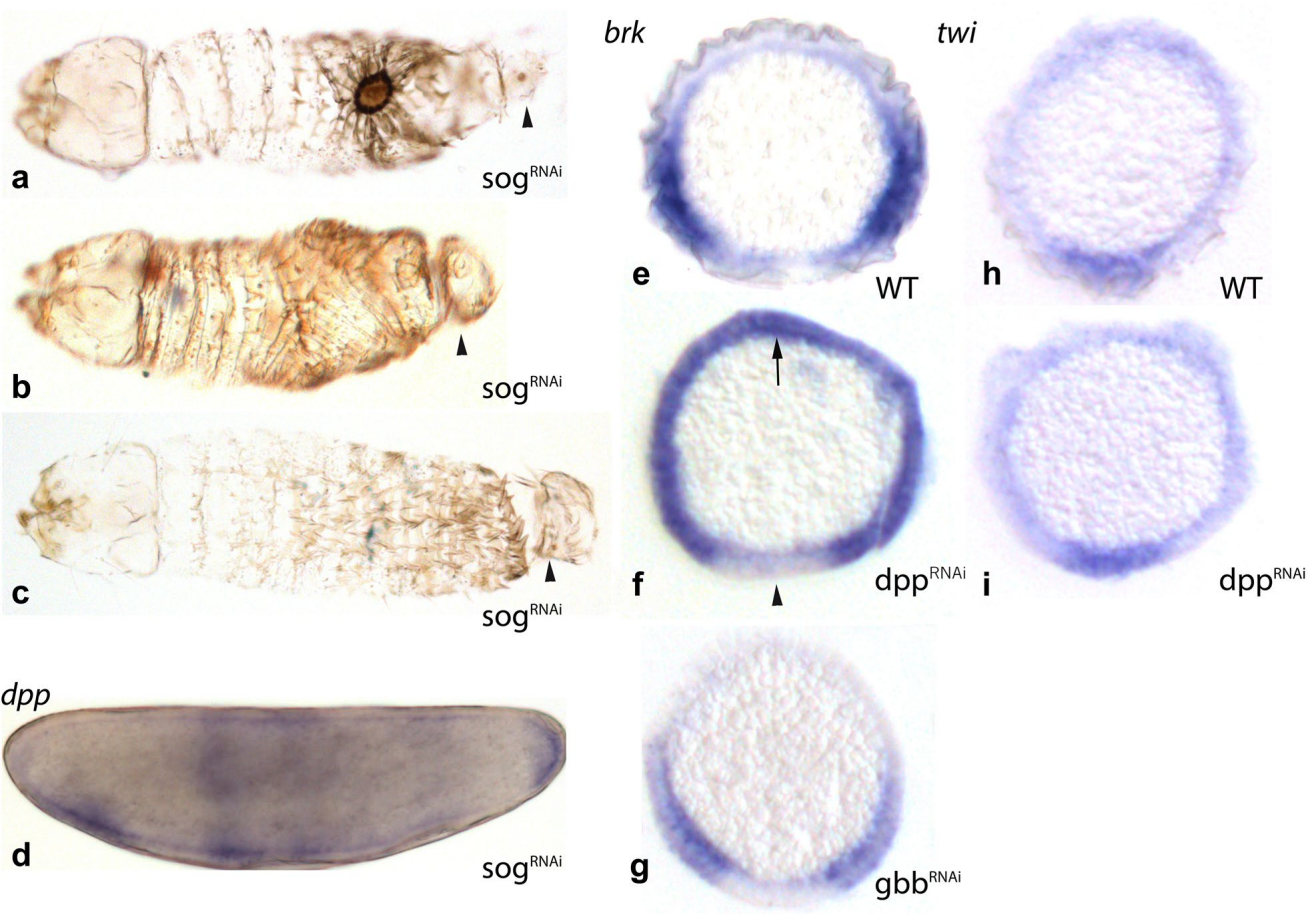
Functional analysis by RNAi knock-down: effects of *sog* and *dpp* RNAi knock-downs in *C. albipunctata*. **a**–**c** Larval cuticle preparations from late-stage embryos treated with *sog* RNAi, exhibiting effects on dorsal closure (**a**), abdominal patterning (**b**, **c**), and terminal patterning (**a**–**c**, arrowheads). Panels d–i show in situ hybridisation (whole-mount, **d**; transverse sections, **e**–**i**). d. Blastoderm-stage embryo treated with *sog* RNAi stained for *dpp* (blue). Embryo shown in lateral view (anterior is to the left, dorsal is up). **e**–**g** Transverse sections of embryos stained for *brk* by in situ hybridisation. **e** Wild type; **f** embryo treated with *dpp* RNAi. Arrow points at dorsal de-repression of *brk*. Arrowhead points at the absence of *brk* expression around the ventral midline; g. embryo treated with *gbb* RNAi. **h**, **i** Transverse sections of embryos stained for *twi* by in situ hybridisation. **h** Wild type; **i** embryo treated with *dpp* RNAi

Attempts to obtain cuticles from *dpp*-depleted embryos failed due to late embryonic lethality. Larval cuticles from *sog*-depleted embryos show severe defects in the abdominal region including one or a combination of the following: failure of dorsal closure (5/16) (Fig. 5a), reduction of the dorsal abdominal region adjacent to the thorax (6/16) (Fig. 5b), compressed abdominal segments (5/16) (Fig. 5c), and constricted or absent terminal region (12/16) (Fig. 5 a–c, arrowheads). Given the nearly abutting expression pattern of *dpp* and *sog*, we wondered whether there was a transcriptional regulatory interaction between the two genes. Such transcriptional effects have been described before in *O. fasciatus* [50], where *sog* is repressed by BMP signalling, as it is in spiders, vertebrates, and sea anemones [51–54]. An effect of *sog* on *dpp* transcription has been reported previously in *Drosophila*, where *sog* blocks *dpp* autoactivation in the neuroectoderm [54], and RNAi knock-down of *sog* in *T. castaneum* leads to downregulation of dorsal expression of *dpp* during segment formation [3]. Although *dpp*-depleted embryos did not show any effects on *sog* (not shown), blastoderm embryos treated with *sog* RNAi show, surprisingly, an expansion of *dpp* expression into the dorsal posterior pole region, plus ectopic expression in the middle of the embryo in 51 out of 73 embryos (Fig. 5d).

To identify the effect of *dpp* knock-down on downstream target genes we stained embryos treated with *dpp* RNAi for markers of neurogenic ectoderm (*brk*) and mesoderm (*twi*). *brk* shows a strong expansion of gene expression dorsally (18/22; Fig. 5f, arrow), indicating an expansion of neurogenic ectodermal tissue to the dorsal midline. In contrast, *brk* expression remains low along the ventral midline (Fig. 5f, arrowhead). Consistent with this, *twi* expression in ventral mesoderm is also not affected (*n* = 18; Fig. 5i). To rule out a redundant role for the other BMP ligand present in *C. albipunctata*, we depleted embryos of *gbb* transcripts using RNAi, but could not detect any discernible effect on *brk* expression compared to the wild type (*n* = 15; Fig. 5g).

## Discussion

### Expression patterns of BMP DV signalling genes in insects

**Fig. 6:**
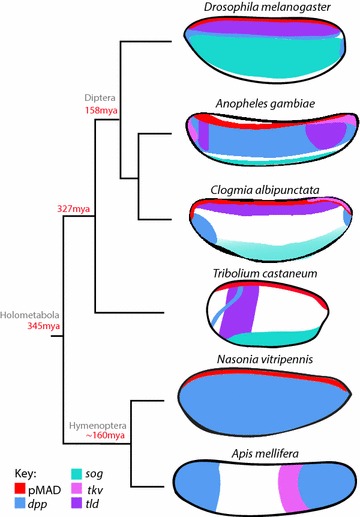
Schematic comparison of localised DV patterning system components in different insects. Embryos are arranged on a phylogenetic tree showing major holometabolan taxa with estimated ages of branching points shown in red (mya: million years ago). Drawings highlight the expression patterns of the principal DV system components in embryos of *N. vitripennis*, *A. mellifera*, *T. castaneum*, *C. albipunctata*, *A. gambiae,* and *D. melanogaster*. Embryos are shown laterally: anterior is to the left, dorsal is up. Red: PMad; blue: *dpp*; green: *sog*; pink: *tkv*; purple: *tld*

Our expression analysis for DV patterning genes in *C. albipunctata* reveals an unexpected amount of upstream variation despite highly conserved target gene output (Fig. 6). Perhaps most surprising is the previously reported restriction of *dpp* expression to two ventral polar domains [25] (Fig. 2a, b), which stands in stark contrast to the dorsal expression domain observed in *D. melanogaster* [15]. This represents an extreme case of the very widespread expression variability for BMP ligands across insect species. In *A. gambiae, dpp* expression is much broader than in *D. melanogaster,* expanding into the ventral–lateral region of the embryo [20]. In *A. mellifera, dpp* is expressed in an anterior and a posterior domain that show no obvious DV polarity [55]. In *N. vitripennis*, *dpp* shows very faint ubiquitous expression before gastrulation [22]. Finally, in early embryos of *T. castaneum, dpp* expression is uniform along the DV axis, with a small posterior cap expression, only becoming restricted dorsally after gastrulation [3, 56]. Outside of the holometabola, in blastoderm embryos of the hemipteran *O. fasciatus dpp* is not detected [50], but is later expressed at the posterior pole, along the dorsal edge of the site of germband invagination [57]. These expression differences may reflect underlying differences in *dpp* regulation. RNAi knock-down of *sog* in *C. albipunctata* leads to expansion of *dpp* expression into the dorsal posterior pole region and ectopic expression in the middle of the embryo (Fig. 5d). Moreover, it leads to abnormalities in the abdominal and posterior patterning as well as dorsal closure (Fig. 5a–c). A transcriptional effect of *sog* on *dpp* has also been described in *D. melanogaster* [54] and in *T. castaneum* [3]. The expansion of *dpp* expression along the AP axis could suggest an implication of the BMP signalling pathway in the AP patterning, as seen in *A. mellifera* [55] and *O. fasciatus* [50]. Such an AP role is further supported by the compression of the abdominal segments in the cuticle phenotypes, although an analysis of the mechanism by which this could occur is beyond the scope of the present study.

A similar amount of variation can be observed for *sog* expression. In *C. albipunctata,* we detect a single ventral expression domain (Fig. 2b–d, g) similar to that seen in *T. castaneum* and *A. gambiae* [3, 20]. In *O. fasciatus*, early blastoderm embryos have ubiquitous expression of *sog*, and only at mid-blastoderm does its expression become ventral, similar to the expression described here [50]. These expression patterns differ from the two medio-lateral domains observed in *D. melanogaster* [58], which therefore may be evolutionarily derived. Interestingly, *sog* appears to have lost its ancestral role in DV patterning in hymenoptera: in *A. mellifera* it is only expressed after gastrulation [4], while in *N. vitripennis* it appears to be completely absent [21].

Other components of the DV patterning system also show variation in expression patterns. In *C. albipunctata,* we observe expression of the ligand *tkv* in a dorsal midline domain, covering the posterior-most ~ 25% of the embryo (Fig. 2e, f). In *D. melanogaster, tkv* is largely, but not exclusively, restricted to the dorsal half of the embryo along the entire antero-posterior axis [59]. In *A. gambiae,* it is initially expressed throughout the dorsal ectoderm, later to be excluded from the presumptive serosa [20]. In *N. vitripennis,* it is faintly expressed ubiquitously [22]. In *A. mellifera,* it is restricted to the posterior third of the embryo, with no DV polarity [55]. This indicates that the localisation of *tkv* expression cannot explain the dorsal localisation of pMAD, as suggested for *D. melanogaster* [60, 61].

Finally, the protease *tld* shows a very dynamic expression pattern in *C. albipunctata.* At the early blastoderm stage, it shows a dorsal expression domain (Fig. 2g), whereas later this expression has completely shifted to the ventral side of the embryo (Fig. 2h). This surprising switch is not seen in any other insect described so far, but expression variations for *tld* in other species are also very striking. Whereas *tld* expression is strictly dorsal in *D. melanogaster* [62], in *N. vitripennis* it is expressed in a small anterior dorsal domain and does not have any function in DV patterning [5]. In *A. gambiae*, *tld* expression is limited to lateral regions and is excluded from the dorsal ectoderm [20]. In *T. castaneum*, *tld* expression occurs in the presumptive germ rudiment and shows the highest levels of expression in a broad anterior domain [24]. In *O. fasciatus*, *tld* is expressed uniformly across the embryo circumference [50].

Taken together, the available evidence reveals a surprisingly large amount of expression variation among upstream signalling factors in the DV patterning system of insects. It is striking that the localisation and extent of expression along the DV axis are not particularly conserved for any of these factors.

### Localised pMAD activity in insect embryos

Compared to the expression patterns of upstream signalling factors, the localisation of pMAD activity is much more conserved across holometabolan insect species. In *C. albipunctata*, pMAD is localised in a narrow stripe along the dorsal midline (Fig. 3a–d), similar to pMAD distributions in *D. melanogaster* (Fig. 3e–g; also see Dorfman and Shilo [63]) and *N. vitripennis* [5]. In *A. gambiae* the dorsal domain of pMAD activity is broader [20]. In *T. castaneum*, pMAD covers the dorsal 50% of the serosa in the anterior, narrowing posteriorly to cover a region of about 20% of the DV axis along the dorsal midline of the germ rudiment [3]. In addition to these similarities, *C. albipunctata* shows a peculiar deviation from the canonical dorsal holometabolan pattern of pMAD. Its DV domain of localisation expands at both anterior and posterior poles of the embryo, to dorsally mirror the ventral polar domains of *dpp* expression (Fig. 3b–d). This polar broadening of the pMAD domain is not seen in any other insect studied so far, although much more subtle polar expansions, predominantly around the posterior pole, are also seen in *Drosophila* and *Megaselia* [40, 64]. The complementary polar patterns of *dpp* and pMAD suggest a correlation between the two. We discuss this observation in the context of a potential BMP ligand shuttling mechanism in *C. albipunctata* below.

### Expression patterns of DV target genes in insects

In *C. albipunctata,* as in other insects, *twi* and *sna* have conserved overlapping ventral domains of expression in the blastoderm embryo [4, 20, 21, 65, 66] (Fig. 4a, b). In *D. melanogaster,* these genes mark the mesodermal anlage [41, 42].

In *D. melanogaster*, DPP signalling represses expression of *brk*, while *brk* in turn is a transcriptional repressor of other DPP target genes [43]. The dorsal morphogen gradient activates *brk* expression in the ventral neurogenic ectoderm, which restricts *dpp* expression to the dorsal half of the embryo [67]. This results in opposite activity gradients of pMAD and *brk* [2]. In *C. albipunctata,* we observe a conserved *brk* expression pattern in two ventral–lateral domains (Fig. 4c), very similar to *D. melanogaster*, *A. gambiae,* and *N. vitripennis* [20, 21, 43]. In contrast, *brk* is not expressed in the early embryo of *T. castaneum* and *sog* alone is responsible for restricting *dpp* expression to the dorsal side of the embryo [3]. It is not clear whether *brk* was recruited independently into DV patterning in hymenopterans and dipterans, or whether it was lost in the coleopteran lineage, although the evidence slightly favours the former scenario (see below).

In *D. melanogaster*, *pnr* and *Doc* are activated by DPP signalling along the dorsal midline of the blastoderm and are involved in dorsal closure and the specification of the extraembryonic amnioserosa, respectively [2, 45, 46]. In *C. albipunctata, Doc* is expressed in a dorsal domain excluding the serosa (Fig. 4d), plus a head stripe, similar to *D. melanogaster*, *A. gambiae,* and late blastoderm embryos of *N. vitripennis* [20, 21]. In *A. gambiae, Doc* expression is restricted to the amnion, but repressed in the serosa [20]. In *T. castaneum, Doc* plays a role in extraembryonic tissue morphogenesis but not specification; it is expressed early in a dorsal anterior domain, then though the entire serosa, but most strongly in its dorsal region [3, 68]. This indicates some variability in the role of *Doc* for determining extraembryonic tissues, which reflects the rapid evolution of these tissues among holometabolan insects [69].

In *D. melanogaster*, *N. vitripennis,* and *T. castaneum*, *pnr* is expressed in a broad dorsal domain during the blastoderm stage [3, 21, 70]. In *C. albipunctata,* it is not detectable until after gastrulation, exhibiting a pattern in the dorsal epidermis and head lobes of the embryo (Fig. 4e). This pattern is similar to that found in *A. mellifera*, where *pnr* shows post-gastrulation expression at the ventral edges of the amnion, resembling post-gastrulation expression in *D. melanogaster* and *N. vitripennis* as well [21]. This indicates that the onset of *pnr* expression seems to vary between species, while its post-gastrulation expression pattern is strongly conserved.

Columnar genes *vnd, ind,* and *msh* are lateral markers of the neurogenic ectoderm: *vnd* is required for the specification of ventral column neuroectoderm, *ind* for the specification of intermediate column neuroectoderm, and *msh* labels all of the dorsal column neuroectoderm [47]. Interaction between DPP and SOG is necessary to restrict the expression of these genes to their respective expression sites [48]. In *N. vitripennis*, like in *D. melanogaster*, these genes are expressed in ventral–lateral stripes [21, 47–49]. In *C. albipunctata*, *ind* and *msh* show similar expression patterns as in other species (Fig. 4g, h). In contrast, *vnd* can only be clearly detected in the head region, with potential additional expression (although faint and diffused) at the posterior pole and the ventral side of the embryo (Fig. 4f). Unlike in *N. vitripennis*, *D. melanogaster, and A. gambiae*, we can only detect expression of all three columnar genes in *C. albipunctata* after gastrulation.

Despite some interesting differences in target gene expression between species, it is evident that downstream DV genes are much more conserved than the upstream signalling factors. In general, we observe a trend towards increasing conservation of expression and localisation patterns as we move downstream in the DV patterning cascade. This parallels the situation in the antero-posterior patterning system, where the most downstream tier of the segment-polarity gene network shows the highest degree of conservation [71–73].

### BMP shuttling in *C. albipunctata* and other insect species

The complex nature of the post-translational shuttling mechanism for BMP ligands poses a challenge for the mechanistic interpretation of our evidence. Comparisons to vertebrate DV patterning suggest that this shuttling mechanism is extremely conserved [10, 17, 74]. What do our data reveal about shuttling of BMP ligands in *C. albipunctata?* The discrepancy between ventral *dpp* expression and dorsal pMAD localisation strongly suggests that some sort of ligand transport must be involved in DV pattern formation in this species.

One important feature that both *D. melanogaster* and *C. albipuncatata* share is the complementarity of their *dpp* and *sog* expression patterns. In *D. melanogaster,* the border between the two domains occurs in the ventral–lateral region of the embryo along the entire length of the antero-posterior axis [58]. In *C. albipunctata,* it is restricted to an anterior and a posterior interface, where *sog* expression abuts, with a slight overlap, the ventrally localised polar *dpp* domains (Fig. 2b). Shuttling is likely to occur at these interfaces as there will be opposing protein gradients of DPP and SOG present at these sites. This suggests that a significant amount of DPP would be shuttled around the anterior and posterior poles of the embryo in *C. albipunctata,* involving transport along the antero-posterior as well as the DV axis, rather than straightforward ventral-to-dorsal transport throughout the embryo as observed in *D. melanogaster.* If our interpretation of the evidence is correct, this can explain the intensified and expanded domains of pMAD in the anterior and posterior polar regions of the *C. albipunctata* blastoderm embryo (Fig. 3b, c).

There are additional differences between the two species. One concerns the posterior-only expression of *tkv* in *C. albipunctata* (Fig. 2e, f). The discrepancy between *tkv* expression and pMAD localisation may indicate that other BMP receptors, such as PUT, may be required for signal transduction in the anterior of the embryo. Even more difficult to explain is the switch of *tld* expression from dorsal to ventral during the late blastoderm stage in *C. albipunctata* (Fig. 2g, h). Its function (if any) remains mysterious, but it either suggests that BMP ligand shuttling must be very dynamic in this species, or that all relevant protein cleavage by TLD and subsequent DPP signalling activity must happen before the dorsal-to-ventral transition in *tld* expression. Further experimental work including immunostaining against the relevant proteins and/or enzymatic activity assays will be required to gain further insight.

A final difference between species is the absence of *scw* in the genome of the non-cyclorrhaphan fly *C. albipunctata* [25]. Comparative analyses suggest that *scw* arose in the cyclorrhaphan lineage from a duplication of the ancestral *gbb* homolog [25, 75]. The SCW ligand is essential for DPP transport in *D. melanogaster* [7]. Is it possible that *gbb* is fulfilling its role in *C. albipunctata? gbb* is required for DPP signalling in *N. vitripennis* [5]. Moreover, it is expressed dorsally in the blastoderm embryo of the scuttle fly *Megaselia abdita* [76], and in a ubiquitous pattern excluding the poles in *C. albipunctata* [25], similar to that seen in *O. fasciatus* [50]. Despite this, our evidence indicates that *gbb* is not needed for DV patterning in *C. albipunctata*. Expression of the target gene *brk* shows no detectable defects in *gbb* knock-down embryos (Fig. 5g), indicating that dorsally localised DPP signalling is occurring correctly in the absence of GBB protein. Taken together, our evidence suggests that DPP is the only BMP ligand contributing to DV patterning in *C. albipunctata.*

Our evidence indicates that BMP shuttling is likely to occur in *C. albipunctata*, although the exact set of factors involved and the spatio-temporal dynamics differ compared to *D. melanogaster*. This further suggests that BMP ligand shuttling is a conserved phenomenon in dipteran insects. The situation is more complicated in other holometabolan taxa. In *T. castaneum,* dorsal localisation of DPP signalling activity depends on SOG and TLD as in flies [3] but does not involve *tsg* [24]. Still, BMP ligand shuttling is probably happening in this species. In contrast, *sog* is not expressed in embryos of *N. vitripennis* [21] and is only expressed at late embryonic stages in *A. mellifera* [4]. Yet, BMP signalling is still responsible for the patterning of the DV axis in *N. vitripennis* [5]. It has been proposed that maternal localisation of BMP receptors, combined with zygotic regulatory feedback, could take the role of dorsal shuttling in hymenoptera [22]. It is not entirely clear whether this condition is ancestral or derived, although the fact that BMP ligand shuttling has been proposed to occur in vertebrates [17] would favour the latter alternative.

Fundamental differences between DV patterning in dipterans and hymenopterans are further supported by the following evidence: RNAi knock-down of *dpp* in *C. albipunctata* leads to an expansion of *brk* expression to the dorsal midline of the embryo (Fig. 5f). This is similar to *brk* expression in *dpp* mutants of *D. melanogaster* [43], but very different to *dpp* knock-down in *N. vitripennis*, where *brk* is restricted to an antero-dorsal expression domain by an otherwise ubiquitous expansion of *twi* [5]. In *A. mellifera*, embryos treated with *dpp* RNAi also show dorsal expansion of *twi* [55], although to a lesser degree than in *N. vitripennis*. In contrast, *C. albipunctata* embryos treated with *dpp* RNAi show wild-type *twi* expression (Fig. 5i), similar to *dpp* mutants in *D. melanogaster* [5] (supporting the difference in the determination of the mesodermal fate by Toll or BMP signalling in dipterans versus hymenopterans). This indicates that *dpp* downregulation in dipterans induces dorsal expansion of the neurogenic ectoderm, while in hymenopterans it leads to an expansion of mesodermal markers. Such fundamental differences in the role of *brk* between dipterans and hymenopterans favour a scenario where *brk* was independently recruited into DV patterning in each lineage [5].

## Conclusions

In this paper, we have analysed the expression of DV patterning factors in the moth midge *C. albipunctata*. A comparison of these expression patterns to those in other insects reveals that expression of upstream signalling factors in the DV system is very variable, while signalling output is highly conserved. This has two major implications.

The first of these two implications is the following: variable expression patterns of upstream factors, in particular the highly unusual ventral expression of *dpp* [25] and the dorsal-to-ventral switch of *tld* in *C. albipunctata* (Fig. 2g, h), complicate the simple traditional picture in which protostomes show dorsal expression of BMP ligands and ventral expression of their inhibitors (e.g. *sog*), while vertebrates show the exact opposite pattern [19]. Our results and other work on BMP DV patterning in insects [3, 5, 20–25, 55] reveal that BMP ligands, as well as other factors involved in their transport, can be expressed at many different positions along the DV axis in different insect species (Fig. 6). This does not argue against the general possibility of a dorsal inversion of the DV axis [18, 19, 77], but indicates that such an inversion would involve more complex spatio-temporal changes in regulation and gene expression than a simple DV switch involving BMP ligands and their inhibitors. As long as ligand shuttling leads to an appropriate localised activation of BMP signalling at either pole of the DV axis, it does not seem to matter all that much where exactly the component factors of the DV system are expressed.

As in the first case, the second implication is also connected to the fact that conservation of gene expression increases for downstream factors in the DV system. This suggests that the DV patterning system evolves by developmental system drift (DSD). DSD is a mode of evolution where a regulatory network gets rewired while maintaining a constant patterning output [33, 34]. It explains that homologous characters can be highly conserved despite substantial divergence in the underlying gene regulatory and signalling mechanisms. DSD has been proposed for many evolving developmental processes such as vulval specification in nematode worms [78], the establishment of left–right asymmetry in vertebrates [79], and the dynamics of antero-posterior patterning in insects [72, 73, 80]. Our results suggest that the early-acting components of the DV patterning system in insects are another strong example of DSD in a generally highly conserved signalling cascade. While most of the examples listed above focus on changes in transcriptional regulation, our work suggests that post-translational regulation—as in transport of BMP ligands—makes important contributions to DSD and will need to be more strongly considered in future studies of the phenomenon.

## Abbreviations

DV: Dorsoventral
BMP: bone morphogenetic proteins
DSD: developmental systems drift
TGF-β: transforming growth factor beta
RNAi: RNA interference
*brk*: *brinker*
*Doc*: *Dorsocross*
*ind*: *intermediate neuroblast defective*
*msh*: *muscle segment homeobox*
*pnr*: *pannier*
*sna*: *snail*
*sog*: *short gastrulation*
*tkv*: *thick veins*
*tld*: *tolloid*
*tsg*: *twisted gastrulation*
*twi*: *twist*
*vnd*: *ventral nervous system defective*
*scw*: *screw*
*gbb*: *glass bottom boat*
*put*: *punt*
*sax*: *saxophone*
MAD: Mothers against dpp
pMad: phosphorylated MAD
BLAST: Basic Local Alignment Search Tool
PCR: polymerase chain reaction
DIG: digoxigenin
FITC: fluorescein
PBS: phosphate-buffered saline
PEMS: pipes, egta, magnesium sulphate, sorbitol
RT: room temperature
PBT: PBS Tween
OCT: optimal cutting temperature
